# Low-protein diets supplemented with casein hydrolysate favor the microbiota and enhance the mucosal humoral immunity in the colon of pigs

**DOI:** 10.1186/s40104-019-0387-9

**Published:** 2019-10-10

**Authors:** Huisong Wang, Junhua Shen, Yu Pi, Kan Gao, Weiyun Zhu

**Affiliations:** 1 Jiangsu Key Laboratory of Gastrointestinal Nutrition and Animal Health, Laboratory of Gastrointestinal Microbiology, College of Animal Science and Technology, Nanjing Agricultural University, Nanjing, 210095 Jiangsu China; 2 National Center for International Research on Animal Gut Nutrition, Nanjing Agricultural University, Nanjing, 210095 Jiangsu China

**Keywords:** Casein hydrolysate, Colonic microbiota, Low-protein diet, Mucosal immunity, Pigs

## Abstract

**Background:**

High-protein diets can increase the colonic health risks. A moderate reduction of dietary crude-protein (CP) level can improve the colonic bacterial community and mucosal immunity of pigs. However, greatly reducing the dietary CP level, even supplemented with all amino acids (AAs), detrimentally affects the colonic health, which may be due to the lack of protein-derived peptides. Therefore, this study evaluated the effects of supplementation of casein hydrolysate (peptide source) in low-protein (LP) diets, in comparison with AAs supplementation, on the colonic microbiota, microbial metabolites and mucosal immunity in pigs, aiming to determine whether a supplementation of casein hydrolysate can improve colonic health under very LP level. Twenty-one pigs (initial BW 19.90 ± 1.00 kg, 63 ± 1 days of age) were assigned to three groups and fed with control diet (16% CP), LP diets (13% CP) supplemented with free AAs (LPA) or casein hydrolysate (LPC) for 4 weeks.

**Results:**

Compared with control diet, LPA and LPC diet decreased the relative abundance of *Streptococcus* and *Escherichia coli*, and LPC diet further decreased the relative abundance of Proteobacteria. LPC diet also increased the relative abundance of *Lactobacillus reuteri*. Both LP diets decreased concentrations of ammonia and cadaverine, and LPC diet also reduced concentrations of putrescine, phenol and indole. Moreover, LPC diet increased total short-chain fatty acid concentration. In comparison with control diet, both LP diets decreased protein expressions of Toll-like receptor-4, nuclear factor-κB, interleukin-1β and tumor necrosis factor-α, and LPC diet further decreased protein expressions of nucleotide-binding oligomerization domain protein-1 and interferon-γ. LPC diet also increased protein expressions of G-protein coupled receptor-43, interleukin-4, transforming growth factor-β, immunoglobulin A and mucin-4, which are indicators for mucosal defense activity.

**Conclusions:**

The results showed that supplementing casein hydrolysate showed beneficial effects on the colonic microbiota and mucosal immunity and barrier function in comparison with supplementing free AAs in LP diets. These findings may provide new framework for future nutritional interventions for colon health in pigs.

**Electronic supplementary material:**

The online version of this article (10.1186/s40104-019-0387-9) contains supplementary material, which is available to authorized users.

## Background

Dietary protein composition can affect the microbiota and mucosal immunity of the large intestine [1]. The colon is the site where there are the greatest number of bacteria and the strongest protein fermentation activity. The colonic mucosa represents the main interacting surface of the gut microbiota and the immune system. Protein fermentation by microbes in the colon can lead to the production of ammonia, amines and indolic and phenolic compounds [2]. Some metabolites such as ammonia, amines, phenol and *p*-cresol are detrimental for the colonic mucosa at the high concentration [2–4]. In pigs, previous studies showed that soybean-based high-protein diets can increase the concentration of ammonia, putrescine, histamine, phenol and *p*-cresol [5, 6], upregulate the gene expression of pro-inflammatory interleukin-8 (*IL-8*) and tumor necrosis factor-α (*TNF-α*) [7], downregulate the protein expression levels of tight junction claudin family members in the colon [8], and even cause diarrhea [9].

Recent researches have indicated that moderately reducing dietary CP levels (≤ 4% reduction compared to National Research Council (NRC) [10]) while supplementing with essential AAs (based on the ideal protein pattern) could reduce proportions of harmful bacteria and protein fermentation products and improve the mucosal immune homeostasis and barrier function in the colon without damaging the growth performance of pigs [11–14]. It is possible that less undigested protein substances in the small intestine enter the large intestine under the low-protein (LP) diet. However, if the level of dietary CP was further reduced (≥ 5% reduction compared to NRC [10], “very low-protein diets”) there were detrimental effects on the colonic microbiota and barrier function of pigs even supplementing the essential and nonessential AAs [12–15]. Although the excessive productions of most protein metabolites are harmful, some of them like amines and indole play important roles in the regulation of normal intestinal functions under the physiological concentration [4, 16]. Indole can enhance the intestinal barrier function and attenuate inflammation [17]. Polyamines, including cadaverine and spermine, have protective effects on intestinal mucosa [18, 19]. Furthermore, short-chain fatty acids (SCFAs), produced by bacterial fermentation of carbohydrate and AAs, can enhance the intestinal barrier function and regulate the mucosal immune homeostasis [20]. With the very LP level, the AA supplementation may nutritionally meet the host requirement, but excessively low level of indole, polyamines or SCFAs due to the insufficient nitrogenous/AA supply for the microbial activity in the colon, may comprise the colonic health.

Dietary proteins and peptides, though highly digestible and easily absorbed in the small intestine, may partly escape digestion in the small intestine and enter into the colon [2]; while free AA may be relatively more absorbed in the small intestine. Moreover, peptides released from the protein do not only provide AAs but also serve as bioactive roles [21]. Peptides are also the preferred substrates for many bacteria probably due to kinetic advantages of peptide-uptake systems [2]. However, it remains unclear that whether the supplementation of peptide in very low-protein diets may be superior to the supplementation of free AAs in regulating the colonic bacterial community and mucosal immunity in pigs.

In the present study, we hypothesized that a supplementation of peptide source, in comparison with free AAs, to the LP diet would benefit the colonic health. To test this hypothesis, we investigated the effect of casein hydrolysate as a peptide source to LP diets, in comparison with free AAs supplementation, on the colonic microbiota and mucosal immunity in pigs. Our results showed benefits of the supplementation of casein hydrolysate to the bacterial community and the humoral immunity in the colon under LP diets. The findings provide new insights into mechanism of dietary peptide effect and important reference for future nutritional intervention for the colon health in pigs fed LP diets.

## Materials and methods

### Experimental design and sample collection

A total of 21 crossbred (Duroc × Landrace × Yokshire, initial body weight 19.90 ± 1.00 kg, 63 ± 1 days of age) pigs from a commercial farm were selected and allowed a 7-day period acclimatization prior to being randomly assigned to 3 treatments, control diet (CP 16%), low protein diet (CP 13%) supplemented with free AAs (LPA), low protein diet (CP 13%) supplemented with casein hydrolysate (LPC) (*n* = 7). All contents of nutrients were similar except for CP, meeting the nutrient requirements of 20–50 kg pigs recommended by the NRC (2012) [10]. The 16% CP level in control diet is a moderately LP level for 20–50 kg pigs according to the NRC (2012) [10] (the recommended CP level is 18%); thus the 13% CP level used in both LP groups is very LP level. Free AAs (Shanghai Kyowa Amino Acid Co., Ltd.) were added to the 3 diets to meet the requirements of standardized ileal digestible (SID) AA of pigs according to the NRC (2012) [10]. In LPC group, casein hydrolysate was supplemented by 4.5% (CP level 89%), replacing the partial AA in LPA diet. The composition and nutrient content of diets, including the composition of AAs, are shown in Table 1. The casein hydrolysate was prepared by casein hydrolysis with food-grade trypsin (Haibo Biotech Inc., Tsingtao, China) and the resulting hydrolyzed casein was subsequently spray-dried to produce a dried powder. Casein hydrolysate contains peptides and AAs in 54 and 46%, respectively.

**Table 1:** Composition and nutrient analysis of experimental diets (as-fed basis)

Items	Control	LPA	LPC
Corn, 8.0%	65.35	50.0	45.0
Soybean meal, 45.2%	18.67	–	–
Casein hydrolysate, 89%	–	–	4.50
Fish meal, 66.1%	1.00	1.00	1.00
Soybean oil	0.72	1.71	2.31
Corn starch	4.00	25.85	25.00
Rice husk powder	0.76	3.54	4.00
Choline chloride, 50%	0.10	0.10	0.10
CaHPO_3_	1.22	1.88	1.99
NaHPO_3_	–	0.26	4.44
*L*-Lys, 78.8%	0.69	1.37	1.00
*DL*-Met, 99%	0.17	0.29	0.18
*L*-Thr, 98.5%	0.29	0.60	0.45
*L*-Trp, 98%	0.07	0.17	0.12
*L*-Leu, 98.5%	–	0.78	0.46
*L*-Ile, 98.5%	0.49	0.53	0.54
*L*-Phe, 98.5%	–	0.53	0.35
*L*-Val, 98.5%	0.19	0.61	0.37
*L*-Asp, 99%	–	0.79	0.56
*L*-Glu, 99%	–	1.45	0.73
*L*-Pro, 99%	–	0.45	–
*L*-Ser, 98.5%	–	0.38	0.20
*L*-Arg, 98.5%	–	0.50	0.20
*L*-His, 80.1%	0.06	0.47	0.20
*L*-Ala, 99%	–	0.37	0.28
*L*-Gly, 99%	–	0.31	0.26
*L*-Tyr, 98.5%	–	0.29	–
*L*-Cys, 98.5%	–	0.26	0.25
Vitamins and minerals premix^a^	1.00	1.00	1.00
Glucose	2.00	2.00	2.00
Sucrose	2.00	2.00	2.00
Stone dust	0.57	0.23	0.10
Cr_2_O_3_	0.35	0.35	0.35
Sodium chloride	0.13	0.14	–
ZnO	0.05	0.05	0.05
Total	100.00	100.00	100.00
Calculated composition			
NE, Mcal/kg	2.41	2.41	2.41
Crude protein, %	16.00	13.00	13.30
Crude fiber, %	2.50	2.50	2.60
Calcium, %	0.70	0.70	0.70
Total phosphorus, %	0.60	0.60	0.63
Available phosphorus, %	0.40	0.49	0.53
Standardized ileal digestible AA, %^b^			
Lys	1.23	1.23	1.23
Met	0.36	0.36	0.36
Ile	0.63	0.63	0.63
Thr	0.73	0.73	0.73
Trp	0.20	0.20	0.20
Val	0.78	0.78	0.78
Leu	1.14	1.23	1.23
Phe	0.64	0.72	0.72
Arg	0.80	0.69	0.51
Gly	0.51	0.46	0.46
His	0.42	0.51	0.39
Cys	0.32	0.32	0.32
Ser	0.62	0.56	0.56
Tyr	0.45	0.42	0.34
Asp	1.17	1.04	1.04
Glu	2.37	2.18	2.18
Pro	0.83	0.78	0.74
Ala	0.68	0.64	0.64

a: Vitamin mixture supplied the following per kg complete diet: vitamin A, 15,000 IU; vitamin D_3_, 3,000 IU; vitamin E, 150 mg; vitamin K_3_, 3 mg; vitamin B_1_, 3 mg; vitamin B_2_, 6 mg; vitamin B_6_, 5 mg; vitamin B_12_, 0.03 mg; niacin, 45 mg; vitamin C, 250 mg; calcium pantothenate, 9 mg; folic acid, 1 mg; biotin, 0.3 mg; choline chloride, 500 mg

b: Values for standardized ileal digestible (SID) concentrations of amino acids for the diets were estimated using standardized ileal digestible coefficients for the various ingredients provided by NRC (2012) [10]

*LPA* Low-protein diets supplemented with free amino acids, *LPC* Low-protein diets supplemented with casein hydrolysate

All pigs were housed individually in stainless steel metabolism cages (2.5 m width × 3.0 m length × 1.6 m height) and fed ad libitum throughout the whole experiment. The pigs had free access to water via a low-pressure nipple drinker. The temperature of the pig house was maintained at 24 ± 2 °C. The pig house and cages were cleaned regularly and the health condition of each animal was closely monitored throughout the experiment. Feed was offered twice daily, and individual feed refusals were recorded. The body weight of pigs was recorded at the beginning and end of the experiment. Then the average daily gain (ADG), average daily feed intake (ADFI) and feed:gain (F:G) were calculated.

The feeding experiment lasted for 28 d. The pigs were slaughtered on d 29 after an overnight fast. The pigs were anesthetized using an intravenous injection of sodium pentobarbital (50 mg/kg body weight) and slaughtered by exsanguination. The intestinal tract was removed immediately after slaughter and colon was identified and ligated before separation. Digesta in proximal colon was collected into sterile tubes and stored at − 80 °C for isolation of bacterial genomic DNA and analysis of SCFAs, lactate, ammonia and biogenic amines. In addition, pH was measured by placing the pH probe within the residual proximal colonic digesta using a portable pH meter. Proximal colonic tissues (about 10 cm from the cecum) of approximately 2-cm in length were collected and then fixed in 4% paraformaldehyde (Sigma, USA) solution for immunohistochemical analyses. Mucosa scrapings were collected by scraping off the mucosa using a sterile glass microscope slide and then stored at − 80 °C for subsequent RNA and protein isolation and immunoglobulin A (IgA) and cytokines detection.

### DNA extraction, PCR amplification and Illumina MiSeq sequencing

Total genomic DNA of bacteria in the colonic digesta was extracted from each sample (0.3 g) using the bead-beating method with a mini-bead beater (Biospec Products, USA), followed by phenol-chloroform extraction [22]. The V3-V4 region of the bacterial 16S rRNA gene was amplified by PCR using bacterial universal primers (341F 5′-AGA GTT TGA TCC TGG CTC AG-3′ and 806R 5′-TTA CCG CGG CTG CTG GCA C-3′). Purified amplicons were pooled in equimolar and 2 × 250 paired-end sequenced on an Illumina MiSeq platform according to the standard protocols at the Majorbio Bio-Pharm Technology (Shanghai, China).

### Colonic bacterial metabolite analysis

Concentrations of SCFAs were determined with gas chromatography according to our previous method [11]. Concentrations of amines and phenolic and indolic compounds were measured with high performance liquid chromatography according to previous methods [23, 24]. The ammonia concentration was analyzed using ultraviolet spectrophotometer [25]. Concentration of lactate was measured by commercially high-sensitivity kits (Nanjing Jiancheng, China) and performed according to the manufacturers’ instructions.

### Gene expression analysis in the colonic mucosa

Real-time quantitative PCR were performed for gene expressions of immune and barrier factors. A list of primers targeting pattern recognition receptors (PRRs), cytokines and barrier function factors can be found in Additional file 1: Table S1. The cytokines were chosen as representative types of T helper 1 cell (Th1) [interleukin-1β (*IL-1β*), *IL-2*, tumor necrosis factor-α (*TNF-α*), interferon-γ (*IFN-γ*), *IL-12 p40*, *IL-18*] or Th2-cytokines (*IL-4*, *IL-5*, *IL-6*, *IL-10*, *IL-13*), which could better represent cellular (mainly mediated by Th1) and humoral (Th2) immune responses [26]. The detailed methods have been described previously [11]. The results were calculated relative to the expression of β-actin with the 2^-ΔΔ^^Ct^ method.

### Immunoblotting of PRRs and immunohistochemistry of mucin-4

To further verify the changes of gene expression of PRRs signaling pathway, we measured the protein levels of Toll-like receptor-4 (TLR4), nucleotide-binding oligomerization domain protein-1 (NOD1), G-protein coupled receptor-43 (GPR43) and nuclear factor-κB (NF-κB) by using western blot analysis as described previously [27]. Total protein was extracted from the colonic mucosa by Tissue Protein Extraction Reagent (78,510, Thermo, USA), and expressions of those indexes were detected using primary antibodies against NF-κB p65 (ab140751, 1:1000; Abcam, UK), TLR4 (ab183459, 1:1000; Abcam), NOD1 (ab97278, 1: 1500; Abcam), GPR43 (ab131003, 1:1000; Abcam) and β-actin (SC-47778, 1:1500; Santa Cruz, USA) and Goat anti-Rabbit IgG (H + L) secondary antibody (31210, 1:5000; Thermo, USA). The quantitative data from western blot bands were expressed as the target protein OD/β-actin OD ratio. Furthermore, colonic mucin-4 (MUC-4) expression was measured by using immunohistochemistry with the primary antibody for MUC-4 (bs-1994R, 1:400; Bioss, USA) and HRP-conjugated secondary antibody (Thermo, USA). The detailed methods have been described previously [28]. The integral optical density (IOD) of each specimen was analyzed by Image Pro Plus 5.0.2. Each sample was used to prepare four slides, and each slide had four sections.

### Cytokine and IgA concentrations analyses in the colonic mucosa

Colonic mucosa samples were homogenized in phosphate buffer solution (1:9, *wt/vol*; pH = 7.4, 4 °C) for 60 s and centrifuged at 3,000×*g* for 20 min at 4 °C, then the supernatant was obtained and used for the determination of cytokines and IgA levels. Protein concentrations of supernatant were detected by using the BCA Protein Assay Kit (Thermo, USA) according to the manufacturer’s instructions. Then concentrations of transforming growth factor-β (TGF-β), TNF-α, IL-1β, IL-10, IL-4, IFN-γ and IgA were measured by enzyme-linked immunosorbent assay (ELISA) according to the manufacturer’s instructions, using commercially high-sensitivity kits [R&D Systems (USA) except for IgA (Bethyl Laboratories, Montgomery, TX)] that are specific for pigs. The levels of cytokines and IgA were expressed as ng/g protein and mg/g protein, respectively.

### Data analysis

Raw data of microbial sequencing were demultiplexed and quality filtered using the QIIME (version 1.17) with the following criteria: the 250 bp reads were truncated at any site receiving an average quality score < 20 over a 50-bp sliding window, exact barcode matching, 2 nucleotide mismatch in primer matching, reads containing ambiguous characters were removed; only sequences that overlap more than 10 bp were assembled according to their overlap sequence. Operational taxonomic units (OTUs) were clustered with 97% similarity cutoff using UPARSE (vsesion 7.1, http://drive5.com/uparse/). Chimeras were checked and excluded using UCHIME. Representative sequences from each OTU were taxonomically classified against the SILVA (Version 128, http://www.arb-silva.de/) database with a confidence level of 80% using the Ribosomal Database Project classifier (version 2.2; http://sourceforge.net/projects/rdp-classifier).

The rarefaction analysis based on Mothur (version 1.31.2, http://www.mothur.org) was conducted to reveal the diversity indices (Chao1 and Shannon). A heat map at genus level was generated using R software (version 3.2.1; https://www.r-project.org/). Principal-coordinate analysis (PCoA) based on unweighted UniFrac distance metrics were performed to visualize the pairwise distances between groups.

Statistical analysis of all data was performed using the SPSS 21.0 (Chicago, USA). The data of growth performance, bacterial metabolites, and mucosal barrier and immune factors were analyzed with the Shapiro-Wilk test for normal distribution detection and then tested for significance by using One-way ANOVA with Tukey’s honestly significant difference. For sequencing data, Kruskal-Wallis test was employed, and *post-hoc* Dunn-Bonferroni test was performed for pairwise comparisons. Then the *P*-values were adjusted with a false discovery rate (FDR) analysis (*q* < 0.05). All data were presented as group mean ± SEM, differences were considered significant at *P* < 0.05.

## Results

### Animal growth performance and pH of colonic digesta

During the whole experimental period, all pigs remained in good health with no clinical signs of diarrhea or health impairment. In comparison with pigs fed on the control and LPA diets, those on the LPC had a greater ADG (*P* < 0.05) (Additional file 1: Table S2). The pH of colonic digesta was lower (*P* < 0.05) in LPC group than that in other two groups (Additional file 1: Table S3).

### Colonic microbial community

**Fig. 1:**
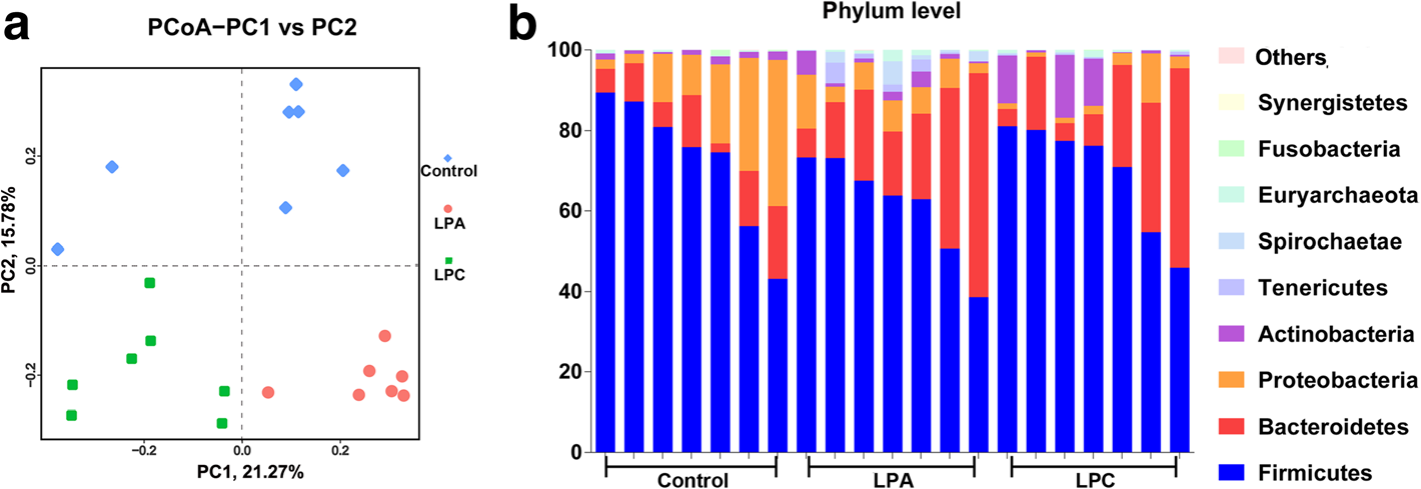
Principle-coordinate analysis (PCoA) of bacterial community by unweighted UniFrac distance (**a**) and distribution (%) of bacteria at phylum level (**b**) in the colon of pigs. In **a**, each point corresponds to an individual animal, and the percentage of variation explained by PC1 and PC2 are indicated in the axis. LPA: Low-protein diets supplemented with free amino acids. LPC: Low-protein diets supplemented with casein hydrolysate

After quality trimming and chimera checking, a total of 1,848,396 16S rRNA gene reads were obtained from each sample. Sequencing depth almost reflected the total microbial species richness (Additional file 1: Figure S1). Whilst there was no difference in the species richness index (Chao1) between the three groups, there was a difference in community diversity (Shannon), with higher (*P* < 0.05) diversity in LPA group than in other two groups. PCoA provided an overview of distinct microbiota composition between each animal (Fig. 1a). PCoA2, accounting for 15.78% of total variance, separated LPA and LPC group from control group. PCoA1, accounting for 21.27% of total variance, separated LPC group from LPA group.

**Fig. 2:**
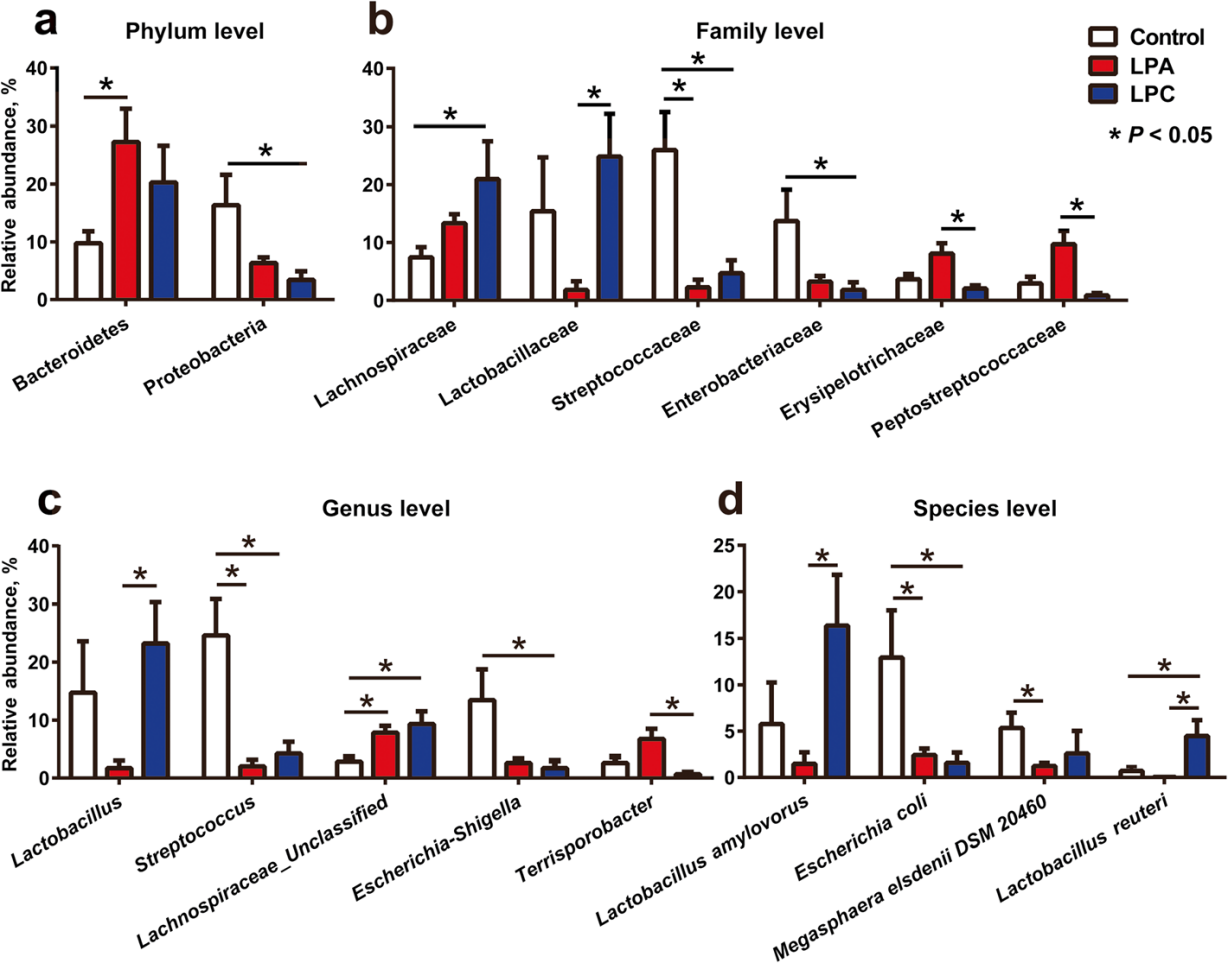
The significantly altered abundance (percentage) of bacterial phyla (**a**), families (**b**), genera (**c**) and species (**d**) in the colon of pigs. Values are means ± SEM (*n* = 7). LPA: Low-protein diets supplemented with free amino acids. LPC: Low-protein diets supplemented with casein hydrolysate

The relative abundances of different phyla are presented in Fig. 1b. The microbiota was dominated by Firmicutes, Bacteroidetes, Proteobacteria and Actinobacteria populations (average relative abundance ≥1%). Among these phyla, LPA diet increased (*P* < 0.05) the abundance of Bacteroidetes, and LPC diet decreased (*P* < 0.05) the abundance of Proteobacteria (Fig. 2a) compared with control diet. At the family level (Fig. 2b), in comparison with control diet, LPA and LPC diet decreased (*P* < 0.05) the abundance of Streptococcaceae, and LPC diet further decreased (*P* < 0.05) the abundance of Enterobacteriaceae and increased (*P* < 0.05) the abundance of Lachnospiraceae. Compared with LPA diet, LPC diet increased (*P* < 0.05) the abundance of Lactobacillaceae and decreased (*P* < 0.05) the abundance of Erysipelotrichaceae and Peptostreptococcaceae. At the genus level (Fig. 2c), in comparison with control diet, LPA and LPC diet decreased (*P* < 0.05) the abundance of *Streptococcus*, and LPC diet further decreased (*P* < 0.05) the abundance of *Escherichia-Shigella*. Compared with LPA diet, LPC diet increased (*P* < 0.05) the abundance of *Lactobacillus* and decreased (*P* < 0.05) the abundance of *Terrisporobacter*. A heat map-based analysis of top 30 genera was shown in Additional file 1: Figure S2.

At the OTUs level, top 30 OTUs were presented in Additional file 1: Table S4. As shown in Fig. 2d, LPC diet increased (*P* < 0.05) the abundance of *Lactobacillus reuteri* compared with other two diets. LPC diet also increased the abundance of *Lactobacillus amylovorus* compared with LPA diet. The abundance of *Escherichia coli* was lower (*P* < 0.05) in LPA and LPC groups than that in control group.

### Colonic microbial fermentation metabolites

**Fig. 3:**
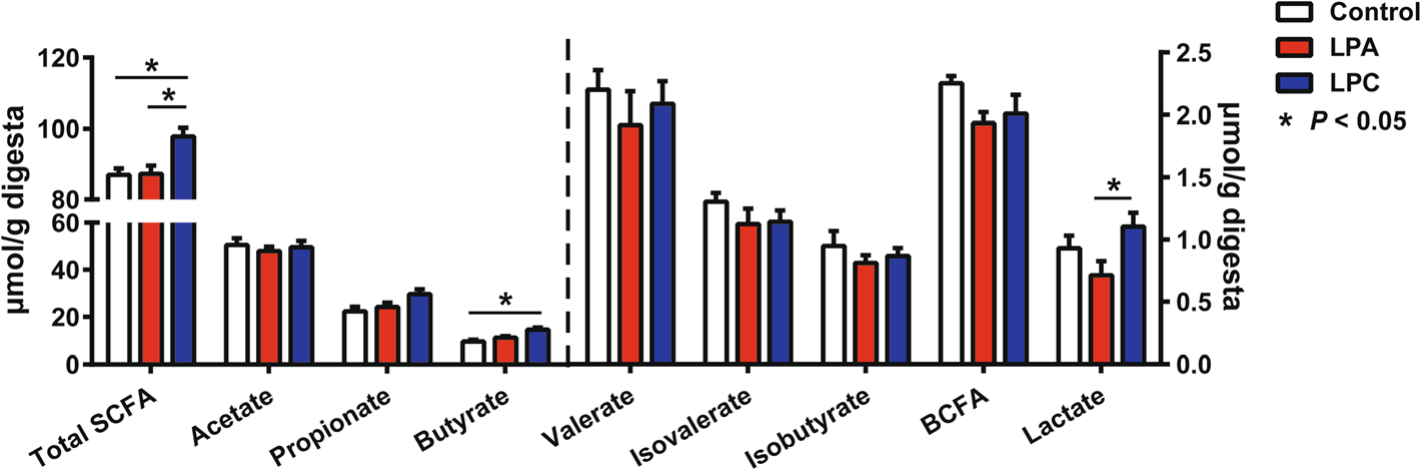
Concentrations of short-chain fatty acids (SCFAs) and lactate in the colonic digesta in pigs. Values are means ± SEM (*n* = 7). BCFA: Branch-chain fatty acid. LPA: Low-protein diets supplemented with free amino acids. LPC: Low-protein diets supplemented with casein hydrolysate

**Fig. 4:**
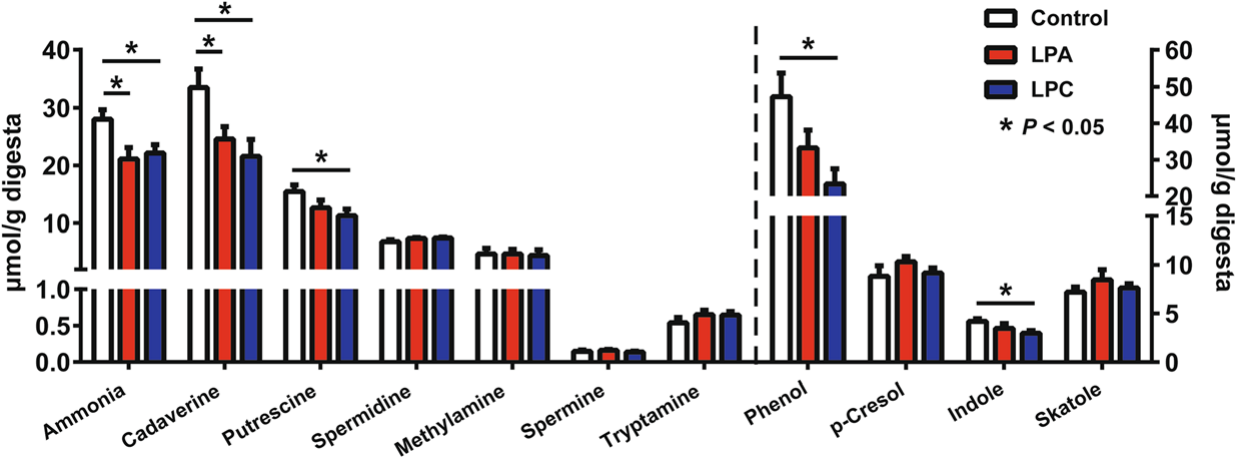
Concentrations of ammonia, amines and phenolic and indolic compounds in the colonic digesta in pigs. Values are means ± SEM (*n* = 7). LPA: Low-protein diets supplemented with free amino acids. LPC: Low-protein diets supplemented with casein hydrolysate

In comparison with the control group, the LPC group showed higher concentrations of total SCFA and butyrate (*P* < 0.05) (Fig. 3). Compared with LPA diet, LPC diet increased (*P* < 0.05) concentrations of lactate and total SCFA (Fig. 3). Ammonia and amines are produced through bacterial deamination and decarboxylation of AAs, respectively. Compared with control diet, both LPA and LPC diets decreased (*P* < 0.05) concentrations of ammonia and cadaverine (Fig. 4), and the LPC diet further decreased (*P* < 0.05) the concentrations of putrescine, phenol and indole (Fig. 4).

### Gene expression levels of colonic immune and barrier factors

**Fig. 5:**
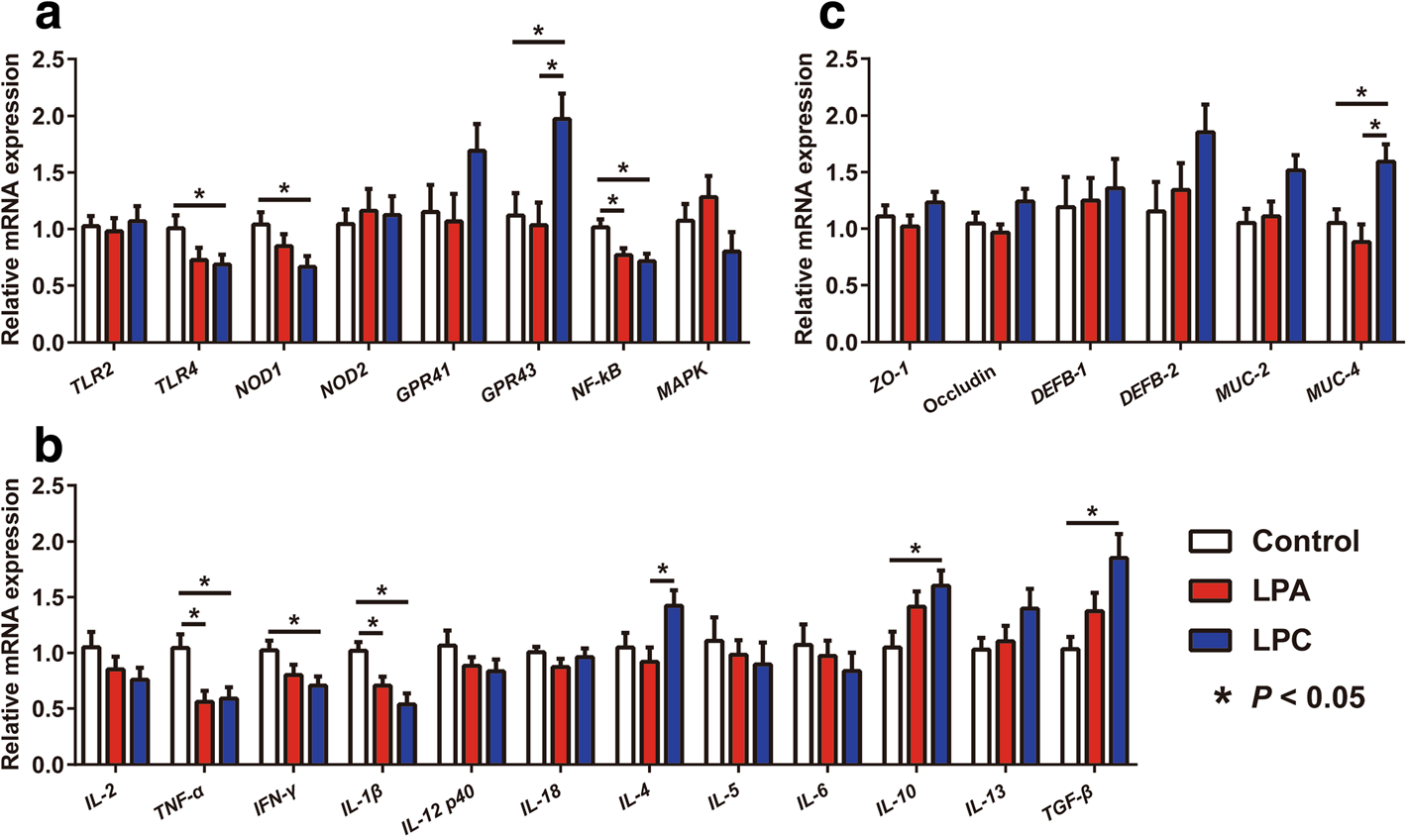
Gene expression levels of colonic mucosal immune (**a**, **b**) and barrier (**c**) factors. Values are means ± SEM (*n* = 7). LPA: Low-protein diets supplemented with free amino acids. LPC: Low-protein diets supplemented with casein hydrolysate. *TLR2*: Toll like receptor 2; *NOD1*: Nucleotide-binding oligomerization domain protein 1; *GPR41*: G-protein coupled receptor 41; *MAPK*: Mitogen-activated protein kinase; *NF-κB*: Nuclear factor-κB; *IL-1β*: Interleukin-1β; *TNF-α*: Tumor necrosis factor-α; *IFN-γ*: Interferon-γ; *TGF-β*: Transforming growth factor-β; *MUC-2*: Mucin-2; *DEFB-1*: β-defensins 1; *ZO-1*: Zonula occludens-1

Both LPA and LPC diets affected the expressions of genes involved in regulating colonic mucosal immune and barrier function (Fig. 5). Compared with control diet, both LPA and LPC diets downregulated (*P* < 0.05) gene expression levels of *NF-κB*, *TNF-α* and *IL-1β*; and LPC diet further downregulated (*P* < 0.05) gene expression levels of *TLR4*, *NOD1* and *IFN-γ*, and upregulated (*P* < 0.05) gene expression levels of *GPR43*, *IL-10* and *TGF-β*. Compared with the LPA diet, the LPC diet upregulated (*P* < 0.05) gene expression levels of *GPR43* and *IL-4*. The LPC diet further upregulated (*P* < 0.05) gene expression level of *MUC-4* compared with other two diets.

### Protein expression levels of colonic immune and barrier factors

**Fig. 6:**
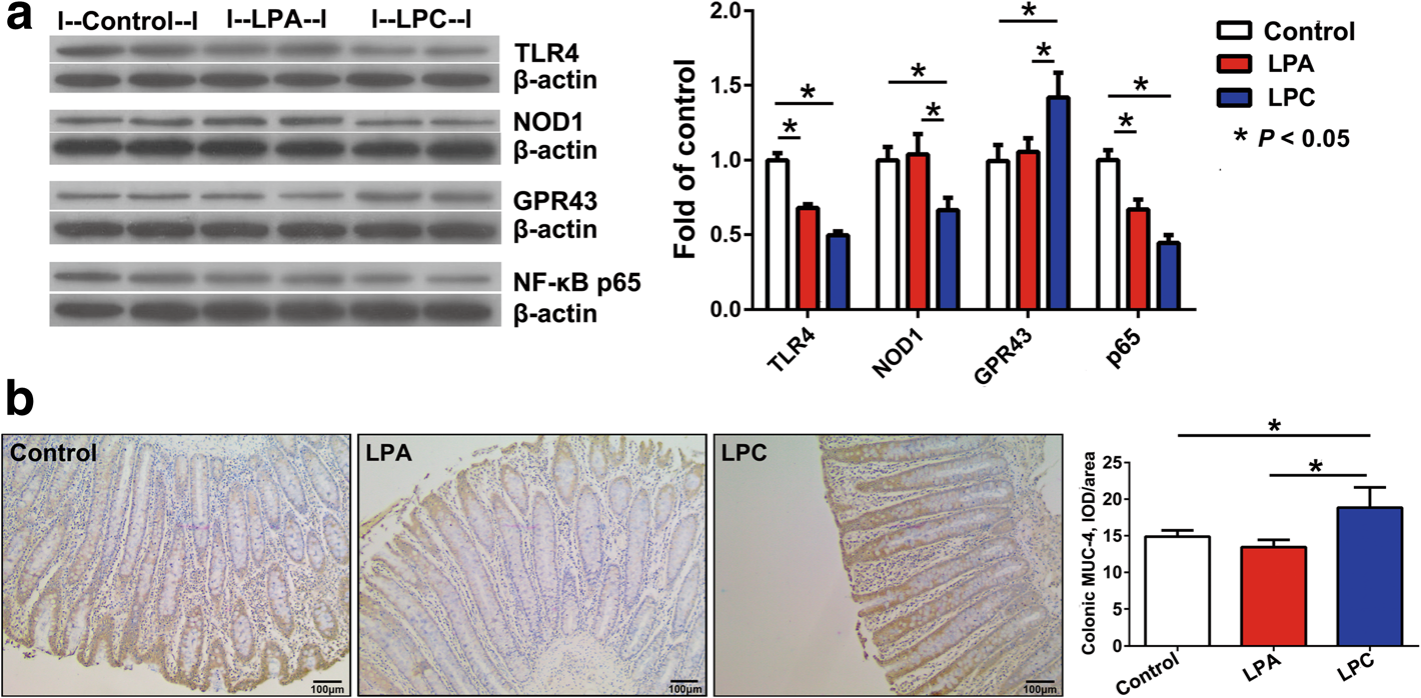
Representative images of staining by immunoblotting and immunohistochemistry in the colon of pigs. **a**, immunoblotting of Toll-like receptor 4 (TLR4), Nucleotide-binding oligomerization domain protein 1 (NOD1), Nuclear factor-κB (NF-κB) p65 and G-protein coupled receptor 43 (GPR43) protein. **b**, immunohistochemical staining of mucin-4 (MUC-4) (magnification: × 100). Values are means ± SEM (*n* = 7). LPA: Low-protein diets supplemented with free amino acids. LPC: Low-protein diets supplemented with casein hydrolysate

Based on the results of gene expressions, we further determined protein expression levels of significantly changed PRRs and cytokines as well as IgA and MUC-4. Compared with control diet, both LPA and LPC diets decreased (*P* < 0.05) protein expression levels of TLR4 and NF-κB p65 (Fig. 6a). Moreover, LPC diet decreased (*P* < 0.05) protein expression level of NOD1, and increased (*P* < 0.05) protein expression level of GPR43 compared with other two diets (Fig. 6a). By using immunohistochemical analysis, we also observed an increased (*P* < 0.05) protein expression level of MUC-4 in LPC group than that in control and LPA groups (Fig. 6b).

**Fig. 7:**
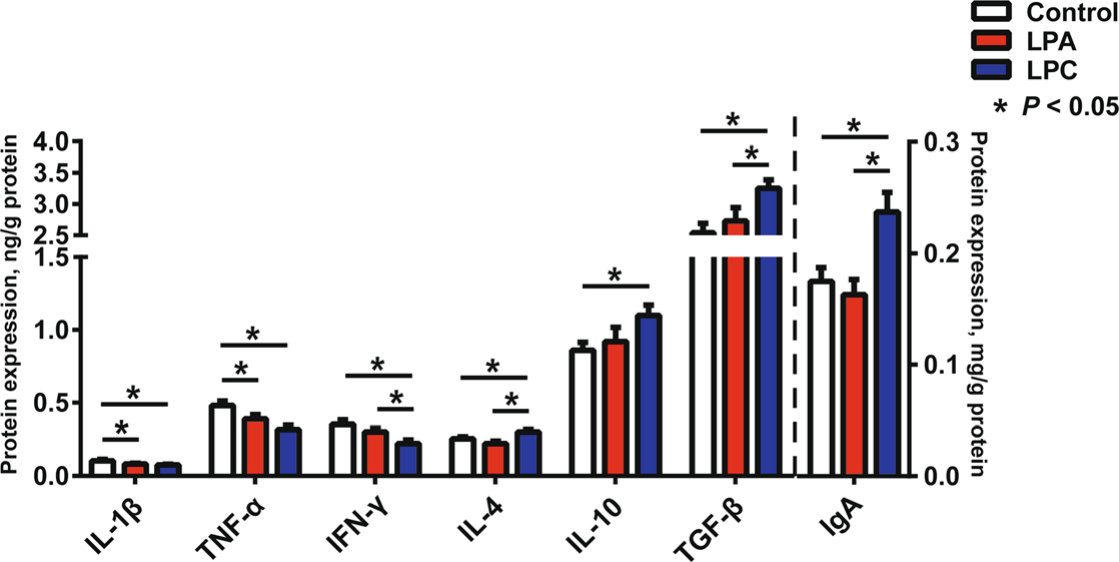
Protein expression levels of colonic mucosal cytokines and IgA in pigs. Values are means ± SEM (*n* = 7). IL-1β: Interleukin-1β; TNF-α: Tumor necrosis factor-α; IFN-γ: Interferon-γ; TGF-β: Transforming growth factor-β. LPA: Low-protein diets supplemented with free amino acids. LPC: Low-protein diets supplemented with casein hydrolysate

In comparison with the control diet, both LPA and LPC diets decreased (*P* < 0.05) protein expression levels of IL-1β and TNF-α, and LPC diet further decreased (*P* < 0.05) protein expression level of IFN-γ, and increased (*P* < 0.05) protein expression levels of IL-4, IL-10 and TGF-β (Fig. 7). Compared with LPA diet, LPC diet increased (*P* < 0.05) protein expression levels of IL-4 and TGF-β and decreased (*P* < 0.05) protein expression level of IFN-γ (Fig. 7). There was a higher (*P* < 0.05) level of IgA in LPC group than in other two groups (Fig. 7).

## Discussion

Previous studies have shown that greatly reducing the dietary CP level detrimentally affects the colonic health in pigs even supplementing the essential and nonessential AAs [12–14], which may be due to the lack of protein-derived peptides. The current experiment compared the effects of casein hydrolysate supplementation and free AA supplementation to the LP diets and showed that casein hydrolysate supplementation to the LP diets further reduced the abundance of potentially pathogenic bacteria and the concentration of nitrogenous metabolites, and increased the abundance of *Lactobacillus*, the concentration of SCFAs and the expression of Th2-cytokines and MUC-4 as compared with AA supplementation. The findings suggest the potential benefits of casein hydrolysate supplementation on the colonic health of the pigs and highlight the importance of protein or protein-derived peptide supplementation in the low-protein diet for the colonic health in pigs.

### Casein hydrolysate supplementation to the low-protein diet (LPC) reduced the abundance of potentially pathogenic bacteria and increased the abundance of *Lactobacillus* as compared with AA supplementation (LPA)

The intestinal bacterial community are significantly affected by the dietary protein composition. In this study, relative to control group, both LPA and LPC diets decreased abundance of potentially pathogenic *Escherichia coli*. But relative to LPA diet, more potentially pathogenic bacteria were reduced by LPC diet, as reflected by the reduction of Erysipelotrichaceae and *Terrisporobacter* in LPC group relative to LPA group, and the reduction of Proteobacteria, including Enterobacteriaceae and *Escherichia-Shigella*, in LPC group, but not in LPA group, relative to control group. Proteobacteria can be seen as microbial signature of dysbiosis in the gut microbiota [29]. The members of Erysipelotrichaceae can promote the development of colitis [30, 31]. *Terrisporobacter* species have been linked to the surgical infection in humans [32]. Therefore, these results suggest beneficial effects of LPC diet, relative to LPA diet, on the colonic bacterial community.

Moreover, relative to LPA group, the enrichment of generally beneficial *Lactobacillus*, including *Lactobacillus amylovorus* and *Lactobacillus reuteri*, in LPC group may further favor the intestinal microbiota. The increase of *Lactobacillus* may be due to that the protein hydrolysates are the preferred substrate for the growth of *Lactobacillus* [2, 33]. Zhang et al. [34] indicated that casein hydrolysate can promote the growth of *Lactobacillus in vitro.* Visser et al. [35] also reported that the supplementation of casein hydrolysate increased counts of fecal *Lactobacillus* in rats.

### LPC diet further reduced nitrogenous metabolites and increased SCFA concentrations as compared with LPA diet

In the colon, dietary proteins and peptides that are undigested in the small intestine can be microbially metabolized to metabolites including ammonia, amines, indoles and phenols [2]. Compared with control group, the reduced concentrations of ammonia and cadaverine in LPA and LPC groups may result from the low flow of undigested CP into the colon, thus reducing the substrate for microbial fermentation. But relative to LPA diet, more nitrogenous metabolites were reduced by LPC diet, including putrescine, phenol and indole. Putrescine is necessary for cancerous colonic epithelial cell mitosis [36]. Phenol can decrease the integrity of the intestinal barrier *in vitro* [37]. But indole has been shown to improve the intestinal barrier function [17]. Therefore, these results suggest that LPC diet, relative to LPA diet, can reduce the concentrations of both deleterious and beneficial protein metabolites.

SCFAs are mainly produced by carbohydrate fermentation by gut microbes. Compared with both the control group and LPA group, the increase in concentration of total SCFA in LPC group indicates an increased bacterial carbohydrate fermentation. Microbes need N substrate and C substrate for cell growth and activity. When the nutrient substrates exceeds the requirement, these nutrients could lead to increased excrements, which is a waste. But an insufficient supply of nutrient substrate could limit the bacterial growth and activity, further limiting the production of SCFAs. As free AAs are easily absorbed in the small intestine, it is possible that with the LPA group supplemented with free AAs, more AAs are absorbed in the small intestine than the control and smaller amount of N entered into the large intestine. This low level of N supply in the large intestine, in comparison with the control group, may lead to a compromised microbial activity including SCFA production by the fermentation of carbohydrate and AAs. This may explain the fact that an extreme reduction of dietary CP level detrimentally affects the colonic health [12, 14, 15]. With the LPC group, under the condition of very LP level, by supplementing the casein peptides, a sufficient level of N may go to the large intestine, which allows microbes to have sufficient activity to produce SCFAs. SCFAs are beneficial on the colonic environment, including but not limited to the suppression of mucosal inflammation [38]. Specifically, butyrate can provide the energy for the colonocyte and enhance the colonic mucosal barrier function [39]. Thus, the increase of total SCFA, including butyrate, in LPC group suggests beneficial effects of LPC diet on the colonic mucosa function. Collectively, these findings on bacterial communities and metabolites suggest beneficial effects of casein hydrolysate supplementation in low-protein diets to the colonic environment.

### LPC diet reduced pro-inflammatory Th1-cytokines expressions and enhanced mucosal potential defense capability as compared with LPA diet

Dietary protein composition, directly or indirectly through gut microbiota, affects the intestinal mucosal immunity [1]. *Escherichia coli* is one of the predominant species in gut and produces lipopolysaccharide (LPS), a major ligand for TLR4. Therefore, in this study, the downregulated expression of TLR4-NF-κB in both LPA and LPC groups may be a reflection of the low proportions of *Escherichia coli*. Our previous study also indicated that there were low gene expression levels of TLR4-NF-κB as results of the reduction of *Escherichia coli* in the colon of pigs fed a LP diet [11]. The changes of TLR4-NF-κB pathway can regulate the expressions of mucosal cytokines. The decreases of pro-inflammatory Th1-cytokine TNF-α and IL-1β in both LPA and LPC groups suggest that Th1-immune responses may be inhibited with both LPA and LPC diets. Th1 responses have been directly related to the onset of intestinal inflammation [40]. The results of the cytokine changes in the present study suggested that both LPA and LPC diets may have the potential in reducing the risk of the intestinal inflammation. Compared with LPA diet, LPC diet further decreased the expressions of pro-inflammatory immune factors, including NOD1 and IFN-γ. NOD1 is an intracellular PRR (intracellular recognition) that predominantly identifies gram-negative pathogens [41]. Thus, the low expression of NOD1 in LPC group may be related to a greater reduction of potential gram-negative pathogens, e.g., *Escherichia-Shigella*, which were decreased in the LPC group. The mechanisms responsible for the greater decrease in the expression of Th1-cytokines in LPC group than LPA group are unknow. Nevertheless, our finding suggests that casein hydrolysate may have the potential in inhibiting the expressions of pro-inflammatory cytokines.

Compared with both control diet and LPA diet, LPC diet enhanced the humoral immunity (mainly mediated by Th2) in the colonic mucosa, as reflected by the increase in expressions of Th2-cytokine IL-4 and IL-10. The increase in IgA expression in the LPC group in comparison of both control group and LPA group also suggests an enhancement of humoral immunity with casein hydrolysate supplementation. TGF-β can activate the maturation of Th2 and B-cell and thus increase the synthesis of IgA [42]. An increase in TGF-β in LPC group further supported the enhancement of humoral immunity. SCFA (acetate, propionate and butyrate) in the colon can promote Th2 differentiation and B-cell maturation [43] as well as Treg-mediated immune response [44]. GPR43, a receptor for SCFA (acetate, propionate and butyrate), can provide a molecular link between SCFA and intestinal mucosal immunity [45, 46]. Therefore, the increase in SCFA concentration may affect the humoral immunity by upregulating the expression of GPR43.

In addition to the enhanced humoral immunity, we also observed a significant increase in MUC-4 expression in LPC group in comparison with control and LPA groups. Casein hydrolysates can promote the secretion of mucins in the gut of mammals [21]. For example, Fernández-Tomé [47] reported that oral casein hydrolysate can stimulate MUC-4 secretion in the rat colon. MUC-4 is a major mucin component constituting colonic mucosa structure and maintaining barrier function [48]. The increased expression of MUC-4 indicates an enhanced colonic mucosal barrier defense in LPC group than LPA group. Collectively, these findings on mucosal immunity and barrier function suggest beneficial effects of casein hydrolysate supplementation in low-protein diets to the colonic mucosal potential defense capability.

### Proposed model of the effects of low-protein diets supplemented with free AAs and casein hydrolysate on the colonic microbiota, microbial metabolites and mucosal immunity

**Fig. 8:**
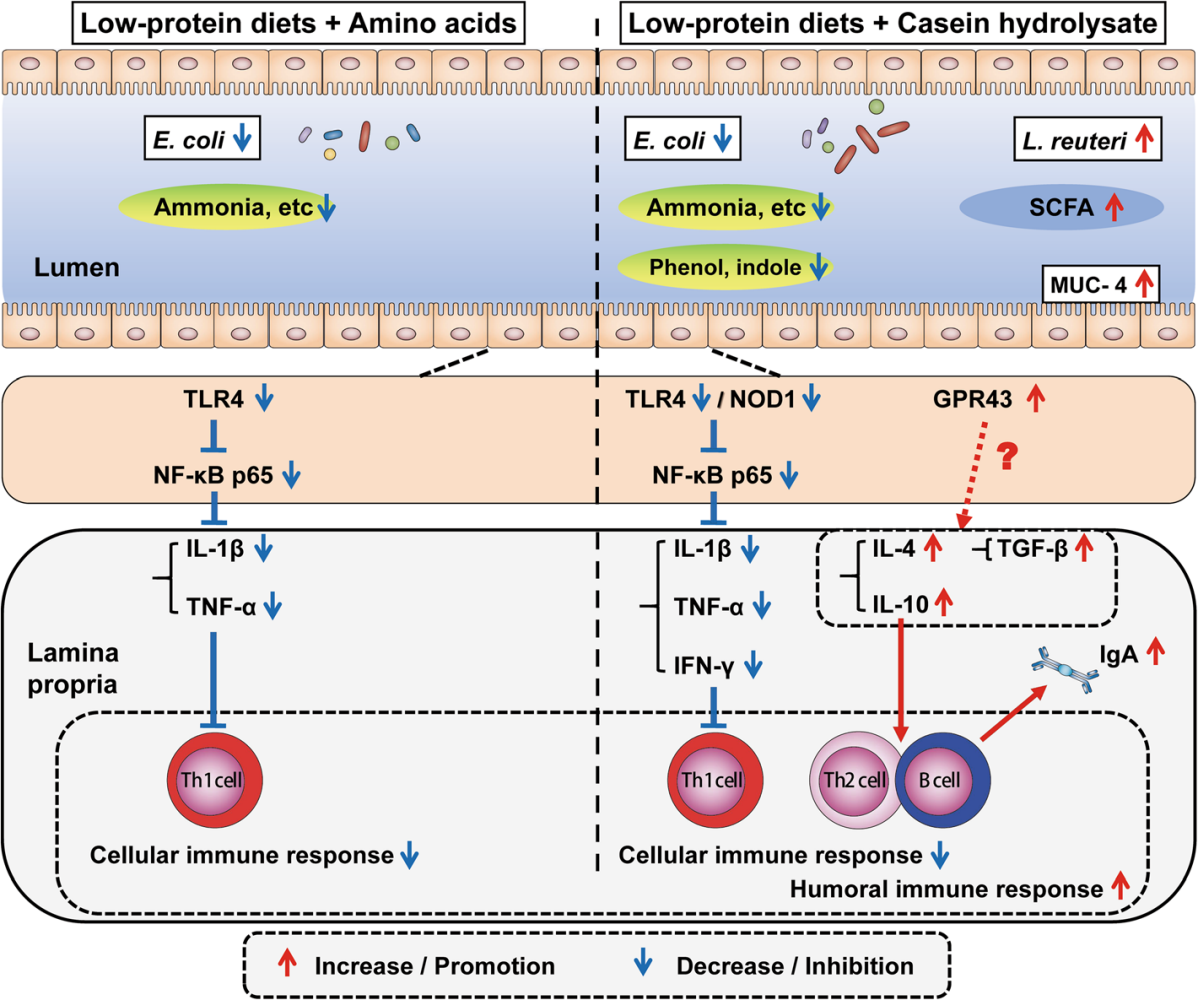
Proposed model of the effects of LPA and LPC diet on gut microbiota, microbial metabolites and mucosal immunity in the colon of pigs. Th: T helper cell; IgA: Immunoglobulin A; IL-1β: Interleukin-1β; TNF-α: Tumor necrosis factor-α; IFN-γ: Interferon-γ; TGF-β: Transforming growth factor-β

Based on our findings, a model of function involved in colonic microbiota, microbial metabolites and mucosal immunity was proposed as shown in Fig. 8. In comparison with control diet, two LP diets, especially LPC diet, decreased the abundance of potential pathogenic bacteria and the concentration of protein fermentation metabolites. LPC diet further increased the abundance of generally beneficial bacteria and the concentration of total SCFA. The cellular immune response was inhibited in both LPA and LPC groups as shown by the decreased expressions of Th1-cytokines. The decreased expression of Th1-cytokines resulted from the inhibited TLR4/NOD1-NF-κB pathway. The humoral immune response mediated by Th2 was enhanced in LPC group as shown by the increased expressions of Th2-cytokines and IgA in the colonic mucosa. GPR43 is upregulated by high level of SCFA, but whether it plays a decisive role in the activation of humoral immunity remains to be explored. Nonetheless, our findings suggest that the supplementation of casein hydrolysate to low crude protein diet is beneficial to the colonic mucosal functions by affecting the barrier functions and immune pathways.

## Conclusions

The colonic microbiota and mucosal immunity in pigs were affected differently by LPA and LPC diets. Compared with LPA diet, the LPC diet decreased the abundance of potentially pathogenic bacteria, the concentration of nitrogenous metabolites and the expression of pro-inflammatory Th1-cytokines. LPC diet further increased the abundance of generally beneficial *Lactobacillus* and the concentration of total SCFA, and enhanced the mucosal potential defense capability with the increased expression of MUC-4 and the activation of humoral immunity. These results suggest a positive effect of the supplementation of casein hydrolysate in LP diets, in comparison with free AAs supplementation, on the colonic microbiota and mucosal immunity in pigs. The findings provide novel insights into nutritional intervention for colon health in pigs, suggesting that a certain quantity of protein-derived peptides are required to favor the colonic environment in pigs fed LP diets.

## Additional file

Additional file 1: **Table S1.** List of primers used in the present study. **Table S2.** The growth performance of pigs. **Table S3.** The pH of colonic digesta and the diversity estimation of 16S rRNA gene libraries from microbiota in the colonic digesta. **Table S4.** The top 30 OTUs in the colonic digesta of pigs. **Figure S1.** Alpha diversity analysis of colonic microbiota. Rarefaction curves for OTUs (a) and rank-abundance curves (b) of average reads. **Figure S2.** Heat-map of top 30 genera in the colonic digesta. (DOCX 233 kb)

## Data Availability

The raw data by pyrosequencing of 16S rRNA genes were subjected to Sequence Read Archive (SRA) database (Accession Number: SRP110931).

## Abbreviations

AAs: Amino acids
ADFI: Average daily feed intake
ADG: Average daily gain
F:G: Feed:Gain
GPR: G-protein coupled receptor
IFN-γ: Interferon-γ
IgA: Immunoglobulin A
IL: Interleukin
LPS: Lipopolysaccharide
MUC: Mucin
NF-κB: Nuclear factor-κB
NOD: Nucleotide-binding oligomerization domain protein
OTUs: Operational taxonomic units
PCoA: Principal-coordinate analysis
PCR: Polymerase chain reaction
PRRs: Pattern recognition receptors
SCFA: Short-chain fatty acid
TGF-β: Transforming growth factor-β
Th: T helper cell
TLR: Toll-like receptor
TNF-α: Tumor necrosis factor-α
Treg: Regulatory T cell
