# Supplementary material for: Low-protein diets supplemented with casein hydrolysate favor the microbiota and enhance the mucosal humoral immunity in the colon of pigs
Source: J Anim Sci Biotechnol. 2019 Oct 10;10:79. doi: 10.1186/s40104-019-0387-9 (PMC6785881; doi:10.1186/s40104-019-0387-9)
Supplement: Supplementary file 1 — Table S1. List of primers used in the present study. Table S2. The growth performance of pigs. Table S3. The pH of colonic digesta and the diversity estimation of 16S rRNA gene libraries from microbiota in the colonic digesta. Table S4. The top 30 OTUs in the colonic digesta of pigs. Figure S1. Alpha diversity analysis of colonic microbiota. Rarefaction curves for OTUs (a) and rank-abundance curves (b) of average reads. Figure S2. Heat-map of top 30 genera in the colonic digesta. (DOCX 233 kb) [file 40104_2019_387_MOESM1_ESM.docx]

**Additional file (Table S1-S4; Fig. S1-S2)**

**Low-protein diets supplemented with casein hydrolysate favor the microbiota and enhance the mucosal humoral immunity in the colon of pigs**

Huisong Wang, Junhua Shen, Yu Pi, Kan Gao, Weiyun Zhu*.

Laboratory of Gastrointestinal Microbiology, Jiangsu Key Laboratory of Gastrointestinal Nutrition and Animal Health, College of Animal Science and Technology, Nanjing Agricultural University, Nanjing 210095, China; National Center for International Research on Animal Gut Nutrition, Nanjing Agricultural University, Nanjing 210095, China.

*Corresponding author: Weiyun Zhu. E-mail: [zhuweiyun@njau.edu.cn](mailto:zhuweiyun@njau.edu.cn)

Address: College of Animal Science and Technology, Nanjing Agricultural University, Nanjing, Jiangsu 210095, China

**Table S1** List of primers used in the present study.

| Target gene | Primer sequences (5'→3') | Reference | Annealing temperature, °C |
| --- | --- | --- | --- |
| *TNF-α* | F:CCACGCTCTTCTGCCTACTGC  R:GCTGTCCCTCGGCTTTGAC | [1] | 60 |
| *IL-1β* | F:AGTGGAGAAGCCGATGAAGA  R:CATTGCACGTTTCAAGGATG | [2] | 60 |
| *IFN-γ* | F:TCCAGCGCAAAGCCATCAGTG  R:ATGCTCTCTGGCCTTGGAACATAGT | [3] | 60 |
| *IL-2* | F:AGCTCTGGAGGGAGTGCTAA  R:TGTTTCAGATCCCTTTAGTTCCA | [4] | 60 |
| *IL-4* | F:GCTGCCCCAGAGAACACGAC  R:AGGTTCCTGTCAAGTCCGCTC | [4] | 60 |
| *IL-10* | F:GTCCGACTCAACGAAGAAGG  R:GCCAGGAAGATCAGGCAATA | [5] | 60 |
| *TGF-β* | F:GAAGATGCTTGGAGCTGAGG  R:TGGGACTTTGTCTTGGGAAC | [5] | 60 |
| *IL-12 p40* | F:GGAGTATAAGAAGTACAGAGTGG  R: GATGTCCCTGATGAAGAAGC | [6] | 60 |
| *IL-18* | F:AGGGACATCAAGCCGTGTTT  R:CGGTCTGAGGTGCATTATCTGA | [7] | 60 |
| *IL-5* | F:TGCCTACGTTAGTGCCATTG  R:TGCCTACGTTAGTGCCATTG | [8] | 60 |
| *IL-6* | F:TGGCTACTGCCTTCCCTACC  R:CAGAGATTTTGCCGAGGATG | [6] | 60 |
| *IL-13* | F:AAGTGGCCCAGTTCGTAAAAGA  R:ACCCGTGGCGAAAAATCA | [7] | 60 |
| β-actin | F:AGAGCGCAAGTACTCCGTGT  R: ACATCTGCTGGAAGGTGGAC | [5] | 60 |
| *GPR43* | F:GTACCTGCCTGGGATCGTCT  R:TGACCACCATGGGGATGAAG | [9] | 60 |
| *GPR41* | F:CTCATCACCAGCTACTGCCG  R:AATTCAGGGTGCTGAGGAGC | [10] | 60 |
| *TLR2* | F:TCACTTGTCTAACTTATCATCCTCTTG  R:TCAGCGAAGGTGTCATTATTGC | [6] | 60 |
| *TLR4* | F:GCCATCGCTGCTAACATCATC  R:CTCATACTCAAAGATACACCATCGG | [6] | 60 |
| *NOD1* | F:ACCGATCCAGTGAGCAGATA  R:AAGTCCACCAGCTCCATGA | [6] | 60 |
| *NOD2* | F:CCTTTTGAAGATGCTGCCTG  R:GATTCTCTGCCCCATCGTAG | [6] | 60 |
| *NF-kB* | F:CTCGCACAAGGAGACATGAA  R:ACTCAGCCGGAAGGCATTAT | [6] | 60 |
| *MAPK* | F:TGCAAGGTCTCTGGAGGAAT  R:CTGAACGTGGTCATCCGTAA | [11] | 60 |
| *ZO-1* | F:*GAGGATGGTCACACCGTGGT*  R:GGAGGATGCTGTTGTCTCGG | [1] | 60 |
| Occludin | F:ATGCTTTCTCAGCCAGCGTA  R:AAG GTTCCATAGCCTCGGTC | [1] | 60 |
| *DEFB-1* | F:ACCGCCTCCTCCTTGTATTC  R:GGTGCCGATCTGTTTCATCT | [6] | 60 |
| *DEFB-2* | F:CTGTCTGCCTCCTCTCTTCC  R:CAGGTCCCTTCAATCCTGTT | [6] | 60 |
| *MUC-2* | F:CTGCTCCGGGTCCTGTGGGA  R:CCCGCTGGCTGGTGCGATAC | [5] | 60 |
| *MUC-4* | F:GTGCCTTGGGTGAGAGGTTA  R:CACTCTGCCGTTCTTTCC | [12] | 60 |

**References**

[1] Zhou X, Kong X, Lian G, Blachier F, Geng M, Yin Y. Dietary supplementation with soybean oligosaccharides increases short-chain fatty acids but decreases protein-derived catabolites in the intestinal luminal content of weaned Huanjiang mini-piglets. Nutr Res. 2014;34:780-8.

[2] Feng Z, Li T, Wu C, Tao L, Blachier F, Yin Y. Monosodium l-glutamate and dietary fat exert opposite effects on the proximal and distal intestinal health in growing pigs. Appl Physiol Nutr Me. 2014;40:353-63.

[3] Villodre Tudela C, Boudry C, Stumpff F, Aschenbach JR, Vahjen W, Zentek J, et al. Down-regulation of monocarboxylate transporter 1 (MCT1) gene expression in the colon of piglets is linked to bacterial protein fermentation and pro-inflammatory cytokine-mediated signalling. Br J Nutr. 2015;113:610-7.

[4] Luo G, Yang L, Liang G, Wan X, Chen C, Wang B, et al. Construction and synergistic effect of recombinant yeast co-expressing pig IL-2/4/6 on immunity of piglets to PRRS vaccination. Procedia in Vaccinology. 2015;9:66-79.

[5] Pieper R, Kroger S, Richter JF, Wang J, Martin L, Bindelle J, et al. Fermentable fiber ameliorates fermentable protein-induced changes in microbial ecology, but not the mucosal response, in the colon of piglets. J Nutr. 2012;142:661-7.

[6] Collado-Romero M, Arce C, Ramírez-Boo M, Carvajal A, Garrido J. Quantitative analysis of the immune response upon Salmonella typhimurium infection along the porcine intestinal gut. Vet Res. 2010;41:23.

[7] Martins R, Lorenzi V, Arce C, Lucena C, Carvajal A, Garrido JJ. Innate and adaptive immune mechanisms are effectively induced in ileal Peyer’s patches of *Salmonella typhimurium* infected pigs. Dev Comp Immunol. 2013;41:100-4.

[8] Williams A, Tva H, Krych L, Ahmad HFB, Nielsen DS, Skovgaard K, et al. Dietary cinnamaldehyde enhances acquisition of specific antibodies following helminth infection in pigs. Vet Immunol Immunopathol. 2017;189:43-52.

[9] Zhang J, Cheng S, Wang Y, Yu X, Li J. Identification and characterization of the free fatty acid receptor 2 (FFA2) and a novel functional FFA2-like receptor (FFA2L) for short-chain fatty acids in pigs: Evidence for the existence of a duplicated FFA2 gene (FFA2L) in some mammalian species. Domest Anim Endocrinol. 2014;47:108-18.e1.

[10] Dong L, Zhong X, He J, Zhang L, Bai K, Xu W, et al. Supplementation of tributyrin improves the growth and intestinal digestive and barrier functions in intrauterine growth-restricted piglets. Clin Nutr. 2016;35:399-407.

[11] Gesslein B, Håkansson G, Carpio R, Gustafsson L, Perez MT, Malmsjö M. Mitogen-activated protein kinases in the porcine retinal arteries and neuroretina following retinal ischemia-reperfusion. Mol Vis. 2010;16:392-407.

[12] Jensen G, Frydendahl K, Svendsen O, Jørgensen C, Cirera S, Fredholm M, et al. Experimental infection with *Escherichia coli O149:F4ac* in weaned piglets. Vet. Microbiol. 2006;115:243-9.

**Table S2** The growth performance of pigs.

| Items | Control | LPA | LPC | *P*-value |
| --- | --- | --- | --- | --- |
| Average daily gain (ADG),  kg/d | 0.55 ± 0.02^a^ | 0.52 ± 0.02^a^ | 0.62 ± 0.01^b^ | 0.020 |
| Average daily feed intake (ADFI), kg/d | 1.24 ± 0.05^ab^ | 1.18 ± 0.04^a^ | 1.35 ± 0.05^b^ | 0.027 |
| Feed:gain (F:G),  kg/kg | 2.35 ± 0.07 | 2.29 ± 0.07 | 2.33 ± 0.09 | 0.790 |

Values are means ± SEMs (n = 7). LPA: Low-protein diets supplemented with free amino acids. LPC: Low-protein diets supplemented with casein hydrolysate.

**Table S3** The pH of colonic digesta and the diversity estimation of 16S rRNA gene libraries from microbiota in the colonic digesta.

| Groups | pH of  colonic digesta | Richness and diversity estimator | |
| --- | --- | --- | --- |
|  |  | Chao1 | Shannon |
| Control | 6.81 ± 0.19^a^ | 226.75 ± 10.76^a^ | 4.11 ± 0.20^a^ |
| LPA | 6.75 ± 0.14^a^ | 285.73 ± 6.69^b^ | 5.73 ± 0.07^b^ |
| LPC | 6.31 ± 0.07^b^ | 243.37 ± 19.02^ab^ | 4.72 ± 0.24^a^ |

Values are means ± SEMs (n = 7). LPA: Low-protein diets supplemented with free amino acids. LPC: Low-protein diets supplemented with casein hydrolysate.

**Table S4** The top 30 OTUs in the colonic digesta of pigs.

| OTU ID | Relative abundance, % | Phylum | Genus |
| --- | --- | --- | --- |
| OTU1 | 13.26 | Firmicutes | *Streptococcus* |
| OTU2 | 9.71 | Firmicutes | *Lactobacillus* |
| OTU3 | 8.29 | Proteobacteria | *Escherichia-Shigella* |
| OTU4 | 6.90 | Firmicutes | *Terrisporobacter* |
| OTU19 | 5.47 | Firmicutes | *Lactobacillus* |
| OTU8 | 5.06 | Firmicutes | *Clostridium sensu stricto 1* |
| OTU7 | 3.93 | Firmicutes | *Megasphaera* |
| OTU28 | 3.65 | Firmicutes | *Phascolarctobacterium* |
| OTU5 | 3.48 | Firmicutes | *[Ruminococcus] torques group* |
| OTU39 | 3.35 | Bacteroidetes | *Prevotella 9* |
| OTU25 | 3.34 | Firmicutes | *Lachnospiraceae_Unclassified* |
| OTU11 | 2.74 | Firmicutes | *Turicibacter* |
| OTU18 | 2.53 | Bacteroidetes | *Prevotella 1* |
| OTU9 | 2.26 | Firmicutes | *Lachnospiraceae_Unclassified* |
| OTU44 | 2.15 | Firmicutes | *Lactobacillus* |
| OTU13 | 2.02 | Firmicutes | *Lachnospiraceae NK4A136 group* |
| OTU23 | 2.00 | Bacteroidetes | *Alloprevotella* |
| OTU47 | 1.78 | Bacteroidetes | *Rikenellaceae RC9 gut group* |
| OTU32 | 1.76 | Bacteroidetes | *Bacteroidales S24-7 group_Unclassified* |
| OTU12 | 1.73 | Actinobacteria | *Bifidobacterium* |
| OTU20 | 1.68 | Firmicutes | *Ruminococcus 2* |
| OTU10 | 1.65 | Actinobacteria | *Collinsella* |
| OTU57 | 1.60 | Firmicutes | *Lachnospiraceae_Unclassified* |
| OTU30 | 1.58 | Bacteroidetes | *Parabacteroides* |
| OTU14 | 1.55 | Firmicutes | *Blautia* |
| OTU15 | 1.55 | Firmicutes | *Ruminococcaceae UCG-005* |
| OTU16 | 1.31 | Firmicutes | *Coprococcus 3* |
| OTU17 | 1.30 | Firmicutes | *[Eubacterium] coprostanoligenes group* |
| OTU6 | 1.29 | Firmicutes | *Subdoligranulum* |
| OTU250 | 1.10 | Firmicutes | *Subdoligranulum* |


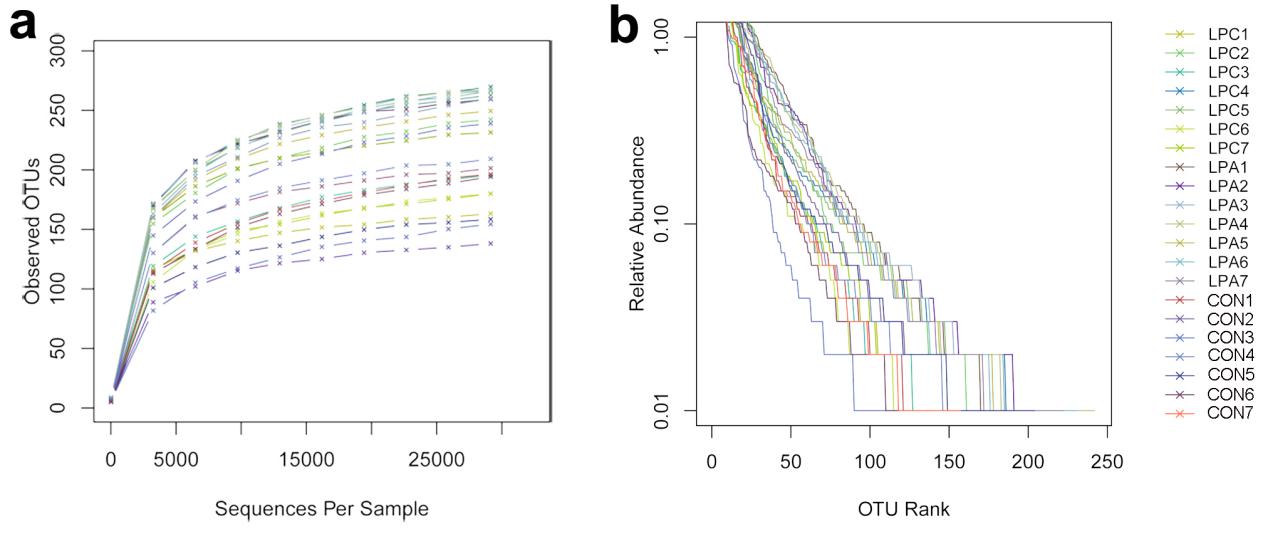


**Fig. S1.** Alpha diversity analysis of colonic microbiota. Rarefaction curves (a) and rank-abundance curves (b) were calculated for reads exhibiting ≥97% sequence identity. CON: Control diet. LPA: Low-protein diets supplemented with free amino acids. LPC: Low-protein diets supplemented with casein hydrolysate.


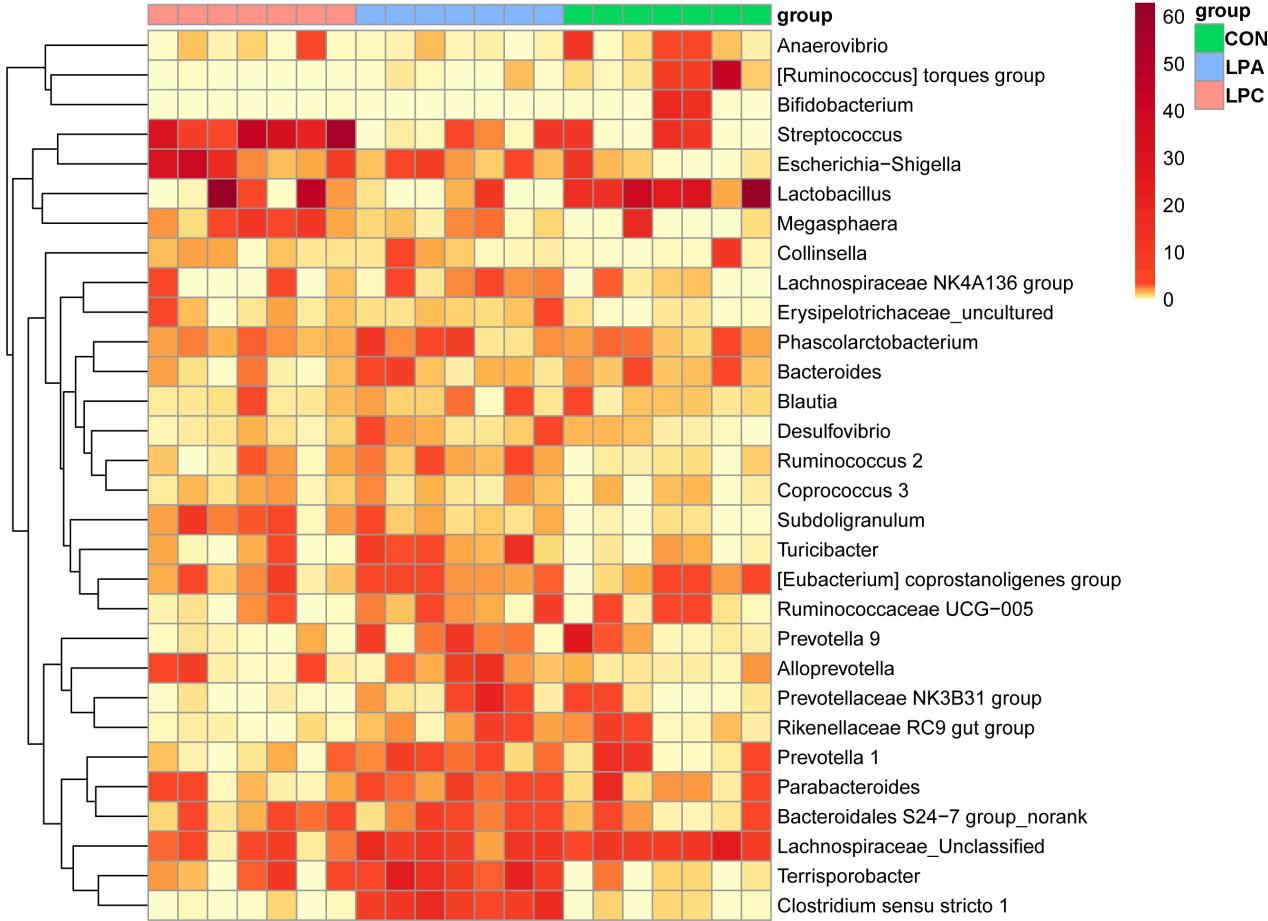


**Fig. S2.** Heat-map of top 30 genera in the colonic digesta. A color gradient showed in the top-right expresses the relative abundance of colonic genera. CON: Control diet. LPA: Low-protein diets supplemented with free amino acids. LPC: Low-protein diets supplemented with casein hydrolysate.
